# Editorial: Altered metabolic traits in gastrointestinal tract cancers

**DOI:** 10.3389/fendo.2024.1390877

**Published:** 2024-05-22

**Authors:** Seema Parte, Ramesh Pothuraju, Ranjith Kumavath, Rakesh Bhatia, Rama Krishna Nimmakayala, Shailendra Gautam

**Affiliations:** ^1^ Department of Biochemistry and Molecular Biology, University of Nebraska Medical Center, Omaha, NE, United States; ^2^ Cancer Research Program, Rajiv Gandhi Centre for Biotechnology (RGCB), Thiruvananthapuram, Kerala, India; ^3^ Department of Biotechnology, School of Life Sciences, Pondicherry University, Puducherry, India; ^4^ Department of Structural Biology, St. Jude Children’s Research Hospital, Memphis, TN, United States; ^5^ Department of Physiology and Cellular Biophysics, Herbert Irving Comprehensive Cancer Center, Columbia University, New York, NY, United States; ^6^ Fred and Pamela Buffett Cancer Center, University of Nebraska Medical Center, Omaha, NE, United States

**Keywords:** gastro-intestinal tract cancers, glycolysis, lipid metabolism, tumor metabolism, oncogenic and metabolic signaling, metabolic targeting

## Introduction

Urban living fosters a sedentary lifestyle, consumption of an unhealthy diet, habitual intake of recreational substances and processed foods. This lifestyle along with genetic susceptibility, environmental pollution and systemic diseases, constitutes a perilous blend that manifests as obesity, metabolic diseases, cardiovascular disorders, and gastrointestinal (GI) cancers (1, 2). In the current era, characterized by the digital revolution, artificial intelligence, and sophisticated machine learning frameworks, predicting future trends in various aspects of life has undergone a significant transformation. However, unraveling the pathological complexities, particularly cancer hidden in the GI tract remains a formidable challenge for the scientific research community considering the alarming death rate. Globally, GI tract cancers are the second- leading cause of cancer-related mortality accounting for 10% of newly registered cancer cases. By 2040, the incidence of GI cancers is anticipated to increase by 1.77 million new cases and 1.27 million deaths worldwide. They pose substantial health and economic challenges due to their multifactorial pathophysiology, lack of early onset symptoms, inaccessible anatomic location, cytotoxicity, low survival rate, lack of appropriate treatment availability, etc., necessitating invasive intervention, the development of undesirable complications, and ultimately oncotherapy failure (3).

**Figure 1:**
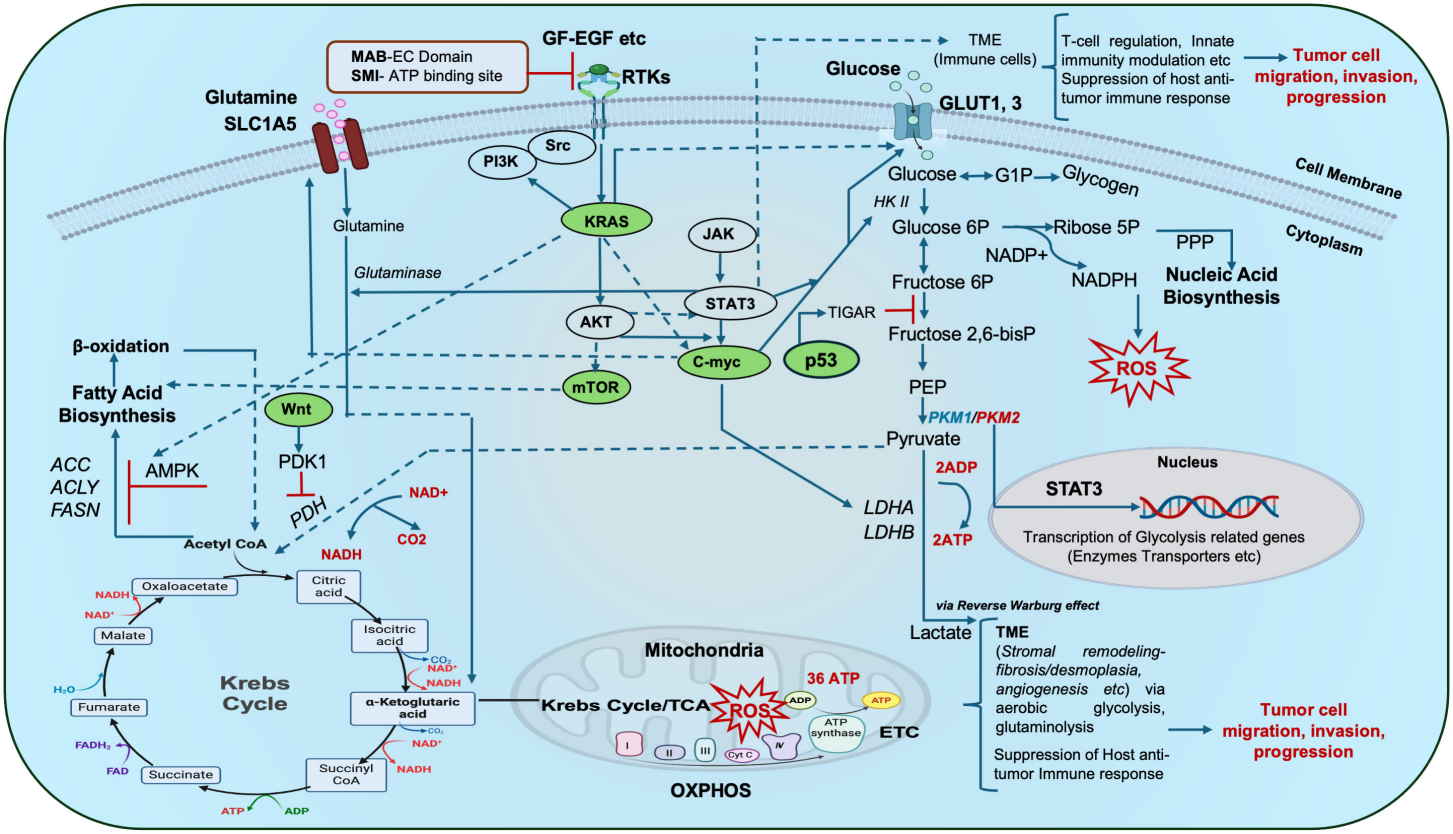
Schematic representation of metabolic hubs that synchronize with key oncogenic/tumor suppressor gene signaling cascades in cancer: The complex interplay of aerobic glycolysis, glutamine metabolism and lipid (fatty acid) metabolism forms a complex repertoire that in turn crosstalks with major oncogenic/tumor suppressor genes and other signaling pathways (Kras, c-myc, p53, mTOR, JAK/STAT3, Wnt etc.) to influence oxidative phosphorylation (OXPHOS), ATP turnover, ROS production, gene transcription etc., and ultimately mitochondrial bioenergetics. Tumor cells with impaired receptor tyrosine kinases (RTKs) reveal altered downstream cellular respiration and signal transduction. Such altered tumor cells may communicate with other cells in their vicinity i.e. the tumor microenvironment (TME i.e. stromal cells, immune cells etc.) during tumor initiation and progression thus complicating their pathophysiology. Oncogenic signaling hubs are indicated in green. Targeting of these RTKs with an antibody-based approach and small-molecule inhibition demonstrate potential targets for onco-therapy. *Dotted portions of lines indicate overlap of the arrows, red bars indicate inhibition. ACC: acetyl CoA carboxylase, ACLY: ATP citrate lyase, ADP-Adenosine diphosphate, AMPK: AMP-activated protein kinase, EGF: Epidermal growth factor, FASN: fatty acid synthase, bisP: bisphosphate, GF: growth factor, G1-P: Glucose 1-phosphate, 6P-6-phosphate, GLUT: Glucose transporter, HKII- Hexokinase 2, LDHA-Lactate dehydrogenase A, MAB EC domain: Monoclonal antibody extracellular domain, NADP/NADPH: Nicotinamide adenine dinucleotide phosphate, OAA: Oxaloacetate, PDH: Pyruvate dehydrogenase, PDK1: Pyruvate dehydrogenase kinase 1, PEP: Phosphoenolpyruvate, PKM1, 2-Pyruvate kinase, PPP: Pentose phosphate pathway, ROS: Reactive oxygen species, RTKs: Receptor tyrosine kinases, SLC1A5: solute carrier family 1 member 5, SMI-Small molecule inhibitor, TCA-Tricarboxylic Acid Cycle, TIGAR: TP53-inducible glycolysis and apoptosis regulator.*.

Compromised receptor tyrosine kinases (RTKs) activate oncogenic signaling pathways such as KRAS, p53, c-Myc, Wnt/β-catenin, PI3K/AKT/mTOR, etc., which are linked with metabolic reprogramming hubs of glycolysis, glutaminolysis, amino acid, nucleic acid, and fatty acid biosynthesis (4). Taken together, dysregulated RTKs, oncogenes and tumor suppressor genes, provoke perturbations in the metabolic circuitry of tumor cells and adjacent ones, either in a cell-autonomous or non-cell-autonomous manner. Mitochondrial biogenesis, respiration and mitophagy are also implicated in tumorigenesis and metastasis. Moreover, oncometabolite accumulation disrupts cellular signaling (**Figure 1**), and the epigenetic machinery aids tumorigenesis. Infections (human cytomegalovirus, helicobacter pylori, etc.), and lymph node metastasis further aggravate GI tumor pathophysiology (4–7).

Investigations are warranted to delineate precise genetic targets and potential signaling pathways while opting for aerobic glycolysis that generates fewer ATP molecules and ROS, but more intermediate metabolites that support anabolic responses and ultimately converge toward enhanced metastatic potential and cancer stemness (8–12). Metabolic restructuring of mitochondrial genes and the protein repertoire impacting tumor hallmarks may be differentially regulated by long non-coding RNAs such as miRNAs. hsa-miR-1343-3p, hsa miR.1228.5p, hsa-miR-5100, hsa-miR-4532, hsa-miR-1290, miR-601, miR-107, miR-18a, miR-370, miR-300, and miR-96 are implicated in GI cancers. Understanding this dysfunctional metabolic activity may enable the development of effective biomarkers for early detection.

This editorial is a collection within the Frontiers in Endocrinology-Cancer Endocrinology section focusing on the theme “Altered metabolic traits in gastro-intestinal tract cancers”, consisting of original research, study protocols and review articles addressing GI tumor metabolism. This consolidated kaleidoscope reveals an interesting relationship between oncogenic signaling and metabolic pathways in GI tumor biology.

## Reviews

Xia et al. reviewed significant aspects of colorectal cancer (CRC) pathogenesis such as gut microbiota, mucosal barrier integrity, immune involvement, signaling cascades (PI3K/AKT, AMPK, mTOR) and differential transcriptional regulation (such as p53, HIF, c-myc, etc.) of glycolysis. Glycolysis-related enzymes and associated signaling pathways play pleiotropic roles in the pathophysiology of GI tumors and have therefore been highlighted as putative therapeutic targets.

Jannin et al. reviewed publicly available gene set enrichments for pancreatic neuroendocrine tumors and identified a Men1 gene mutation that regulates oxidative phosphorylation and glycolysis. Transcriptomic and metabolomic approaches revealed dysregulated one-carbon metabolism, glutathione metabolism, fatty acid biosynthesis, oxidation, and branched-chain amino acid catabolism replenishing the tricarboxylic acid cycle, and mTOR and unfolded protein responses collectively favoring tumor cell growth. Further investigation of potential downstream targets or biomarkers is required.

In the current Research Topic, Li et al. highlighted lipid regulatory factors and epigenetic machinery, synchronous with oncogenic signaling pathways that conspire to deregulate lipid metabolism thus contributing to tumor development. They emphasized a symbiotic metabolism between heterogeneous cells within the tumor microenvironment (TME) and tumor cells. Studies suggest targeting lipid metabolism in addition to conventional therapy as a potentially effective approach.

## Study protocols

The repercussions of impaired metabolic machinery, resulting in aggressive tumor spread, and oncotherapy-related side effects such as cachexia and cardiovascular damage-oriented complications have an undesirable impact, necessitating the rationalization of onco- and palliative therapy. In this Research Topic, the findings of Malta and Goncaveles revealed the pathophysiology of cachexia as a relevant hazard for GI malignancies, compromising the postoperative health and quality of life of patients. They proposed that grape seed flour extract modulates inflammation and oxidative stress during disease management thus positively impacting treatment outcomes.

## Original research

Chen et al. investigated the combination of aspirin and the natural flavonoid vitexin as an effective treatment method for CRC. Drug-disease-target network construction analysis revealed key signaling pathways (NFKB1, PTGS2, MAPK1, MAPK3 and TP53) as downstream gene targets of combination therapy. *In vitro* drug combination assays demonstrated ternary complex formation by simultaneous docking to Cox2 and NFKB, thus inhibiting CRC cell proliferation compared to a mono-drug approach. Therefore, further *in vitro*, and preclinical animal model studies are required.

In summary, this Research Topic includes studies on metabolic activities, with emphasis on glucose and lipid metabolic irregularities, and their profound impact on GI tumor biology. Remarkably, in silico and *in vitro* tests suggested a combination therapy (vitexin and aspirin) for GI tract cancers, thus providing an opportunity for drug repurposing. A randomized clinical trial demonstrated the impact of polyphenol-rich grapeseed flour on weight loss and cachexia associated with CRC by modulating inflammation and oxidative stress. The editorial also links several perturbed metabolic pathways to the pathophysiology of pancreatic neuroendocrine tumors, thereby providing a more comprehensive understanding of these intricate malignancies.

## Future perspectives

Innovative immunomodulatory methods and multiomics-based patient profiling can streamline the development of precise therapies by targeting glycolysis-linked enzymes, regulators, and signals through small-molecule inhibition, CRISPR-based RNA silencing or gene editing. The epigenetic regulation of metabolic enzyme functions and the collective influence of heterogeneous metabolic profiles in non-tumor cells, including TME, on tumor cell metabolism remain largely unexplored. Future strategies that simultaneously target cancer hallmarks (e.g., immune evasion, metastasis, resistance to oncological treatment, etc.) may help reduce GI cancer-related morbidity and mortality. Hence, personalized cancer therapies and precision oncology, which take into account individual complexities and the intricate metabolic dynamics between tumor cells, tumor-initiating stem cells and TME, are indeed the need of the hour.

## Author contributions

SP: Conceptualization, Data curation, Formal analysis, Software, Writing – original draft, Writing – review & editing, Supervision, Visualization. RP: Formal analysis, Writing – original draft, Writing – review & editing, Conceptualization. RK: Conceptualization, Formal analysis, Writing – review & editing. RB: Conceptualization, Formal analysis, Writing – review & editing. RN: Conceptualization, Formal analysis, Writing – review & editing. SG: Conceptualization, Formal analysis, Writing – review & editing.
